# Sublingual Administration of Sildenafil Oro-dispersible Film: New Profiles of Drug Tolerability and Pharmacokinetics for PDE5 Inhibitors

**DOI:** 10.3389/fphar.2018.00059

**Published:** 2018-02-06

**Authors:** Luca De Toni, Maurizio De Rocco Ponce, Erica Franceschinis, Stefano Dall’Acqua, Roberto Padrini, Nicola Realdon, Andrea Garolla, Carlo Foresta

**Affiliations:** ^1^Department of Medicine, Unit of Andrology and Reproductive Medicine, University of Padova, Padova, Italy; ^2^PharmaTeG - Pharmaceutical Technology Group, Department of Pharmaceutical and Pharmacological Sciences, School of Medicine, University of Padova, Padova, Italy; ^3^Department of Pharmaceutical and Pharmacological Sciences, School of Medicine, University of Padova, Padova, Italy; ^4^Clinical Pharmacology Unit, Department of Medicine, School of Medicine, University of Padova, Padova, Italy

**Keywords:** PDE5 inhibitors, drug formulation, sublingual route, erectile dysfunction, adverse drug reaction

## Abstract

**Objective:** Type 5 phosphodiesterase inhibitors (PDE5i) are efficient drugs used for treatment of erectile dysfunction (ED); however, a large discontinuation rate due to major side effects is reported. The aim of this study was to evaluate the possible improvement of sildenafil (Sild) pharmacokinetics associated to the sublingual administration of the new available oro-dispersible film (ODF), compared to both the oro-dispersible tablet (ODT) and the film-coated tablet (FCT) as original *per os* formulation.

**Methods:** *In vitro* disaggregation test, dissolution test, and permeation test in specific devices to estimate the trans-mucosal absorption. *In vivo* analysis of serum Sild levels, by high performance liquid chromatography-tandem mass spectrometry (HPLC-MS/MS), was performed in 20 patients with psychogenic ED receiving alternatively *per os* FCT or sublingual ODT or ODF, at an equal dosage (50 mg). Pharmacokinetic parameters of Sild and adverse drug reactions experienced after the dosing of each formulation were compared.

**Results:** *In vitro*, ODF showed the highest time to disaggregation and an increased rate of permeation compared to both ODT and FCT (*P* = 0.017 and *P* = 0.008, respectively). *In vivo*, compared to both FCT and ODT, ODF showed a faster increase of serum Sild levels (serum levels at 15 min from dosing, respectively: 2.24 ± 1.4 ng/ml FCT, 0.5 ± 0.3 ng/ml ODT, and 13.5 ± 9.1 ng/ml ODF; *P* < 0.01 and *P* < 0.05 vs. ODF) together with a higher drug bioavailability within 60 min from dosing (relative AUC_60__min_ vs. FCT, respectively: 100.0 ± 44.9% FCT, 183.8 ± 75.4% ODT, and 304.2 ± 156.0% ODF). A trend toward lower peak serum levels was observed for ODF. Finally, ODF showed a lower prevalence of headache compared to FCT (1 vs. 35%; *P* < 0.05) and improved pattern of flushing and nasal congestion.

**Conclusion:** Sublingual Sild ODF improves the drug tolerability through a likely modified pharmacokinetic, suggesting a possible implication also in the clinical efficacy profile. Sublingual administration of oro-dispersible formulations may represent a strategy to ameliorate the adherence to therapy with PDE5i, particularly in patients discouraged by side effects.

## Introduction

The enzymatic action of type 5 phosphodiesterase (PDE5) is the primary mechanism for inactivation of cGMP, the downstream mediator of the vasodilating agent nitric oxide (NO) (Lugnier, 2006).

In course of erectile dysfunction (ED), the impaired production of NO from the endothelial cavernous vessels can be pharmacologically overcome by the use of PDE5 inhibitors (PDE5i), in order to prolong the cGMP half-life and to enhance the residual vasodilating function (Hawksworth and Burnett, 2015). Several molecules with inhibitory activity on PDE5 have been then projected and released on the market, showing different onset and duration of effect. Currently, PDE5i are the first-choice drugs used for the treatment of ED (Mehrotra et al., 2007).

Despite a recognized efficacy in nearly 80% of unselected ED patients (Eardley et al., 2010; Hatzimouratidis et al., 2010; Porst et al., 2013), a remarkable drop-out from the treatment with PDE5i has been recently reported. In quantitative terms, an average discontinuation rate of 4% per month has been reported, with an overall abandonment of therapy in 50% cases on an annual basis (Carvalheira et al., 2012; Corona et al., 2016). Among the main reasons given by patients to justify the abandonment of therapy, the lack of efficacy and side effects are referred as the most prevalent (Corona et al., 2016). There can be distinguished two kinds of side effects: those strictly related to PDE5 inhibition, such as headache, flushing, and dyspepsia, and those associated to residual inhibitory activity of drugs on other PDE, such as vasodilation and tachycardia (PDE1), visual disturbances (PDE6), and back pain (PDE11) (Bischoff, 2004; Gupta et al., 2005). Since the occurrence of side effects increases with both serum levels and time exposure to the drug (Gupta et al., 2005; Taylor et al., 2009), the safety/efficacy profile of a drug can be alternatively improved through a pharmacokinetic approach by the design of a novel drug formulation (Mehrotra et al., 2007). This approach has been successfully applied to PDE5i in the case of Vardenafil. Indeed, this molecule suffers of a relatively low bioavailability (∼15%, Center for Drug Evaluation and Research, 2003). Compared to the film-coated tablet (FCT), the original formulation for classical *per os* administration, the formulation of oro-dispersible tablets (ODT) for Vardenafil significantly increased the drugs bioavailability by favoring the sublingual absorption, a route acknowledged to be less affected by first-pass metabolism (Heinig et al., 2011).

Sildenafil (Sild) was the first selective PDE5 inhibitor approved for the treatment of ED (Boolell et al., 1996). It is a relatively lipophilic molecule and, after oral administration, the peak plasma concentration is achieved in a time varying from 0.5 to 2 h. Sild displays a relatively low oral bioavailability (38–41%), mainly due to extensive gut and first-pass metabolism (Gupta et al., 2005). Despite the long presence on the market, the development of novel formulations of drug has poorly pursued, until the recent release on the Italian market of a new formulation: the oro-dispersible film (ODF). Indeed, ODF was approved as a bioequivalent form of FCT (Leoni et al., 2013). However, orally disintegrating formulations may represent suitable systems to favor trans-mucosal, and in particular sub-lingual, absorption (Kathpalia and Gupte, 2013).

In this study, we evaluated the Sild pharmacokinetics associated to sublingual administration of either ODF or ODT, in comparison to the FCT as original *per os* formulation. To this aim, we investigated the release/permeation profile of the different Sild formulations by *in vitro* systems specifically developed to evaluate the trans-mucosal absorption of drugs. Furthermore, we quantified the serum profiles of Sild pharmacokinetics after the administration of *per os* FCT, and sublingual ODT and ODF in patients with ED.

## Materials and Methods

### Chemicals and Drug Formulations

Hank’s balance salts solution pH 7.4 (HBSS), HEPES solution, benzanilide, and Sild citrate were all purchased from Sigma–Aldrich (Milan, Italy). In order to avoid any confounding results deriving from the use of alternative bioequivalent products available on the market, the following products were used: Viagra^®^ FCT (Pfizer, Milan, Italy), Viagra ORO^®^ ODT (Pfizer), and Rabestrom^®^ ODF (IBSA, Lodi, Italy). Drugs were prescribed to patients during outpatient evaluation for their private use. For *in vitro* tests and *in vivo* evaluation of pharmacokinetics (see below), drugs were specifically purchased by personnel involved in the study at local pharmacy facilities and then handled/stored in optimal conditions.

### Disaggregation Test

The *in vitro* disaggregation tests were performed according to European Pharmacopoeia (2016) using the Tablet Disintegration Tester (Sotax DT 2, **Supplementary Figure S1A**), using water as immersion fluid at 37 ± 0.5°C. The time of disaggregation was checked at complete disintegration of dosage form. Complete disintegration is defined as that state in which any residue of the unit remaining on the screen of the test apparatus or adhering to the lower surface of the discs is a soft mass having no palpably firm core. Tests were performed in triplicate and results were reported as mean value ± standard deviation.

### Dissolution Test

*In vitro* drug dissolution tests were performed according to European Pharmacopeia [using dissolution apparatus 2 (Sotax AT7 Smart, **Supplementary Figure S1B**)]. The dissolution tests were performed using a paddle apparatus, paddle speed 50 rpm, and HBSS pH 7.4 was used as dissolution medium volume (900 ml at 37 ± 0.5°C).

During the release tests, 2 ml of dissolution medium sample, at 0, 2, 4, 6, 10, 20, 30, and 40 min, was removed and filtered through 0.45 μm cellulose esters filter and hence diluted. Subsequently, Sild quantification was performed by high performance liquid chromatography (HPLC)-UV. The removed volume was replaced each time with fresh medium. Tests were performed in triplicate and results were reported as mean value ± standard deviation. Data were normalized on the drug content of the formulation.

### Trans-mucosal Permeation Test

Trans-mucosal permeation profile of Sild was evaluated *in vitro* by using a specific device according to Delvadia et al. (2012) appropriately modified. Briefly, the device consisted in a vertical diffusion system featured by donor–receiver chambers separated by a disposable cellulose–acetate membrane (pore 0.45 μm). The donor cell contained 1.5 ml of HBSS, whereas the receiver chamber was part of a closed recirculation circuit of 30 ml including the volumes of tubing and reservoir. Sampling was performed from receiver chamber reservoir at the time intervals of 0, 5, 10, 20, 30, and 40 min and replaced by an equivalent volume of fresh HBSS. The samples were analyzed by HPLC-UV for drug content (Fejős et al., 2014). Data were normalized on the highest permeated Sild concentration observed for pure Sild citrate. Tests were performed in triplicate and results were reported as mean value ± standard deviation.

### Pharmacokinetic Study on Volunteer Psychogenic ED Patients

The study was conducted in the Unit of Andrology and Reproductive Medicine (University Hospital of Padova, Italy), between May and September 2017, according to the Declaration of Helsinki under the approval of the Ethics Committee of the Padova University Hospital (protocol number 3982/AO/16 and successive amendments). In order to avoid confounding results, subjects with psychogenic ED were enrolled because the lower occurrence of organic derangements associate to ED (Ludwig and Phillips, 2014).

The sample size was calculated in order to attain an effect size of at least 0.5 with a statistical power of 0.8 and a significance level of 0.05 for a three group comparison (see power calculator – one-way independent ANOVA).

Twenty patients (mean age 31.4 ± 5.7 years) were consecutively enrolled by the release of signed informed consent. Patients attended outpatient evaluation reporting the consistent inability to obtain and maintain an erection for satisfactory sexual intercourse during the previous 6 months or more. Subsequent clinical evaluation was performed in all patients in order to ascertain the absence of diseases related to ED, such as diabetes mellitus, hypertension, neurological disorder, and the use of antidepressants. Diagnosis of psychogenic ED was confirmed by the fulfillment of an index score <26 at the administration of the International Index of Erectile Function-15 (IIEF-15) questionnaire (Lotti et al., 2016) and the maintenance of nocturnal spontaneous erections, assessed by Nocturnal Penile Tumescence and Rigidity Monitoring through the RigiScan Plus Rigidity Assessment System (Dacomed, United States) for two consecutive nights as previously described (Munoz et al., 1993; Caretta et al., 2005). Exclusion criteria were diagnosis of malignancies, abnormal hormone plasma levels (respectively, luteinizing hormone >8 UI/l, total testosterone <10.4 nmol/l, thyrotropin >4.5 mUI/l, prolactin >20 ng/ml), and increased intima-media thickness at supra-aortic trunks (>0.9 mm) and/or cavernous arteries (>0.3 mm), assessed by Color Doppler-Ultrasound as previously described (Caretta et al., 2005, 2009).

**FIGURE 1:**
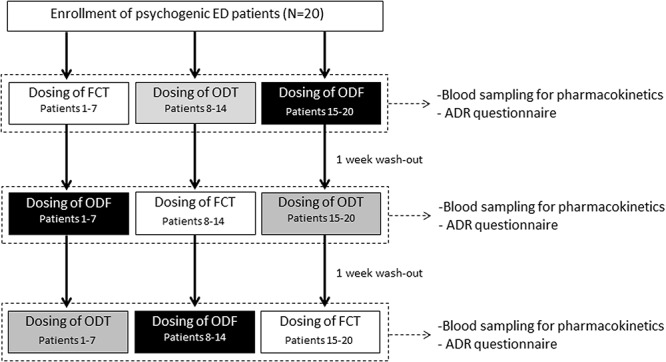
Dosing scheme and assessments for the evaluation of pharmacokinetic parameters in 20 patients with psychogenic erectile dysfunction (ED), subdivided into three groups (respectively, Patients 1–7; Patients 8–14; and Patients 15–20). Each group of patients alternatively received *per os* film-coated tablet (FCT), sublingual oro-dispersible tablet (ODT), or sublingual oro-dispersible film (ODF) with at least 1 week of wash-out from each dosing. All formulations contained 50 mg of sildenafil (Sild).

Patients agreed to adhere to a single dose, three-way crossover open-label study. Drug dosing scheme is reported in **Figure 1**. The dosage of Sild was chosen as 50 mg, in agreement with previous reports on the treatment of psychogenic ED (Banner and Anderson, 2007). Patients were requested to take no drugs or alcohol for 1 week prior to and during the course of the study. Furthermore, during the day of drug dosing, patients were also requested to have a non-fat breakfast (no milk or other fat food) at least 2 h before the test. FCT, considered as reference formulation, was classically administered *per os* by swallowing the oral tablet with a glass of water. For ODT and ODF, patients were instructed to hold the formulation under the tongue for 15 min without the assumption of water, followed by swallowing. Venous blood samples were collected in standard tubes (Vacutainer, BD Biosciences, Milan, Italy) at 0, 5, 10, 15, 30, 60, 90, 120, and 240 min after drug administration. Compensation through continuous infusion of sterile saline solution was performed. After the dosing, subjects were housed until completing the blood sampling. An additional blood withdrawal was performed at 24 h from dosing for control reasons to ascertain the complete drug clearance. As washout periods, 7 days were allowed between one dosing and the following. After blood collection, plasma was immediately isolated and stored at -80°C until use.

During the housing, patients were also requested to record adverse drug reactions (ADR) in a specifically conceived form, adapted from the ADR reporting form of the Agenzia Italiana del Farmaco (AIFA^1^). A translated version of the questionnaire is available as Supplementary Material (see **Supplementary Data Sheet S1**). Patients were asked to specify the type of adverse reaction/s, the overall intensity (in a subjective assessment scale from 1, very weak, to 5, very intense), the time of onset, and overall duration.

### Quantification of Sildenafil in Human Serum

For the quantification of serum levels of Sild, 400 μl of serum sample was added of 500 μl of methanol supplemented of the internal standard (IS) benzanilide (5 μg/ml). Subsequently, 5 ml of ethyl acetate was added to the mixture, the samples were vortexed to perform liquid–liquid extraction, the two phases were separated by centrifugation. The organic portion was then evaporated under vacuum-centrifuge at 40°C. The residue obtained was re-dissolved with 200 μl of methanol and finally used for high performance liquid chromatography-tandem mass spectrometry (HPLC-MS/MS) analysis.

High performance liquid chromatography-MS/MS analysis used a system Agilent-Varian 1260 and the triple-quadruple detector Agilent-Varian 320 MS. Chromatographic separation was performed on a column Phenomenex C-18 Evo 3 × 100 [Phenomenex, Castel Maggiore (BO), Italy] and a mobile phase constituted of acetonitrile with 0.1% formic acid in water with a gradient detailed in **Supplementary Figure S2A**. A positive detection-mode was applied for the transition 198 > 105 Da of the IS, obtained with a capillary tension of 40 V and a collision energy of 16 V while 475 > 100 Da for Sild with capillary tension of 40 V and a collision energy of 22.5 V, respectively. Representative chromatograms of the IS and Sild in real samples are reported in **Supplementary Figure S2B**.

### Statistical Analysis

Pharmacokinetic parameters (PK), such as maximal serum concentration (*C*_max_), time to maximal serum concentration (*t*_max_), and area under the curve (AUC) at different time points, were calculated with specific routines of applications with GraphPad software (La Jolla, CA, United States). Statistical analysis of data was performed with SPSS 21.0 for Windows (SPSS, Chicago, IL, United States). The Kolmogorov–Smirnov test was used to check for normality of distribution. Variables not showing normal distribution were log transformed. Baseline characteristics of patients, PKs, and data from *in vitro* experiments were compared with unpaired Student’s *t*-tests with Bonferroni–Holm correction for multiple comparisons. Repeated-measures ANOVA was performed to test differences in drug release and Sild serum concentration. Levene’s test was used to test the homogeneity of variance among groups. If homogeneity of variance assumption was violated, Welch test was performed and the respective *P*-value was reported. The proportion of ADR was compared with χ^2^ exact test. *P*-values < 0.05 were considered as statistically significant.

## Results

### *In Vitro* Analysis of Release/Permeation of Sildenafil from the Different Drug Formulations

**FIGURE 2:**
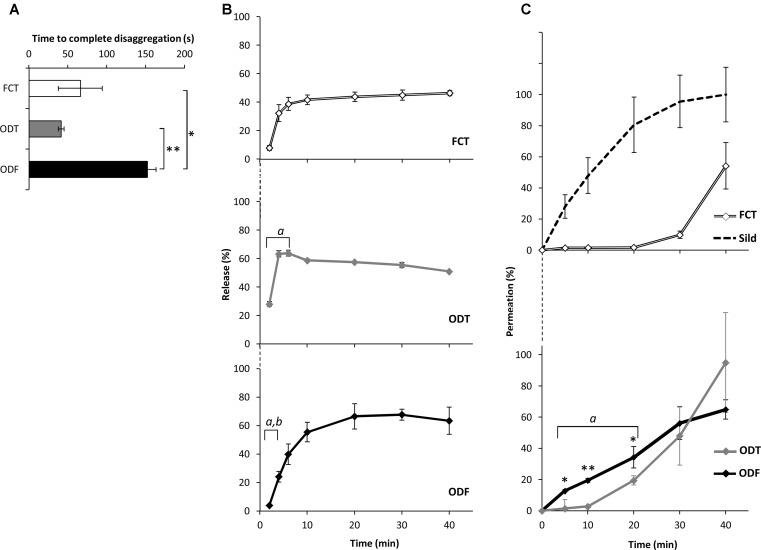
*In vitro* evaluation of bio-technological properties of FCTs, ODTs, andODFs containing 50 mg of Sild. Results are representative of three independent experiments and reported as mean values ± standard deviation. **(A)** Results of the disaggregation test, reporting the time (in seconds) to the complete disaggregation of the formulation. Significance: ^∗^*P* < 0.05 and ^∗∗^*P* < 0.01 between the indicated formulations. **(B)** Results of the dissolution test, reporting the amount of Sild released by each formulation as percentage of the drug’s dosage. Significance: *^a^P* < 0.01 vs. FCT; *^b^P* < 0.001 vs. ODT. **(C)** Results of the permeation test (detailed in the section “Materials and Methods”) reporting the amount of Sild permeated through a cellulose acetate membrane, as percentage of the highest value achieved by 50 mg of pure Sild powder (discontinuous line in the upper panel). Significance: ^∗^*P* < 0.05 vs. ODT; ^∗∗^*P* < 0.01 vs. ODT; *^a^P* < 0.01 vs. FCT.

The dynamic of release and permeation of Sild from the three drug formulations was formerly investigated *in vitro* (**Figure 2**). The time to complete disaggregation for FCT, ODT, and ODF was evaluated through a standard test described in the European Pharmacopoeia (2016; **Figure 2A**). ODF showed the highest time to disaggregation compared to both FCT and ODT (respectively, *P* = 0.017 and *P* = 0.008).

In order to evaluate the influence of this evidence on the drug release from the formulation, a standard dissolution test was performed (European Pharmacopoeia, 2016; **Figure 2B**). Compared to the reference formulation FCT, ODT showed a faster release of the drug whose maximal extent was achieved even after only 2 min from the beginning of the test (*P* = 0.006 vs. FCT). On the other hand, the percentage of the drug released from ODF within the first 4 min of the assay was lower compared to both FCT and ODT (respectively, *P* = 0.004 and *P* < 0.001). In particular, ODF showed a more progressive release of the drug compared to FCT, achieving the maximal extent after 20 min from the beginning of the test.

On this basis, the trans-mucosal permeation of Sild from each formulation was assessed by a specifically developed device (**Figure 2C**). For reference purposes, pure Sild citrate, used in a weight equivalent to 50 mg of Sild, was assesses for trans-mucosal permeation and showed free and fast diffusion between the two chambers. An almost opposed profile was observed for FCT, showing a negligible permeation until 30 min from the beginning of the test. The permeation profiles of ODT and ODF showed an intermediate behavior compared to the two previous conditions. Within the first 20 min of the assay, ODF showed a significantly higher extent of permeation compared to ODT (respectively, *P* = 0.033 at 5 min, *P* = 0.003 at 10 min, and *P* = 0.041 at 20 min). Differently from FCT and ODT that completely disaggregated in the donor chamber, a residual soft-jelly mass of ODF persisted on cellulose acetate membrane even at the end of the assay (**Supplementary Figure S2C**).

### Differential Pharmacokinetics of Sildenafil

**FIGURE 3:**
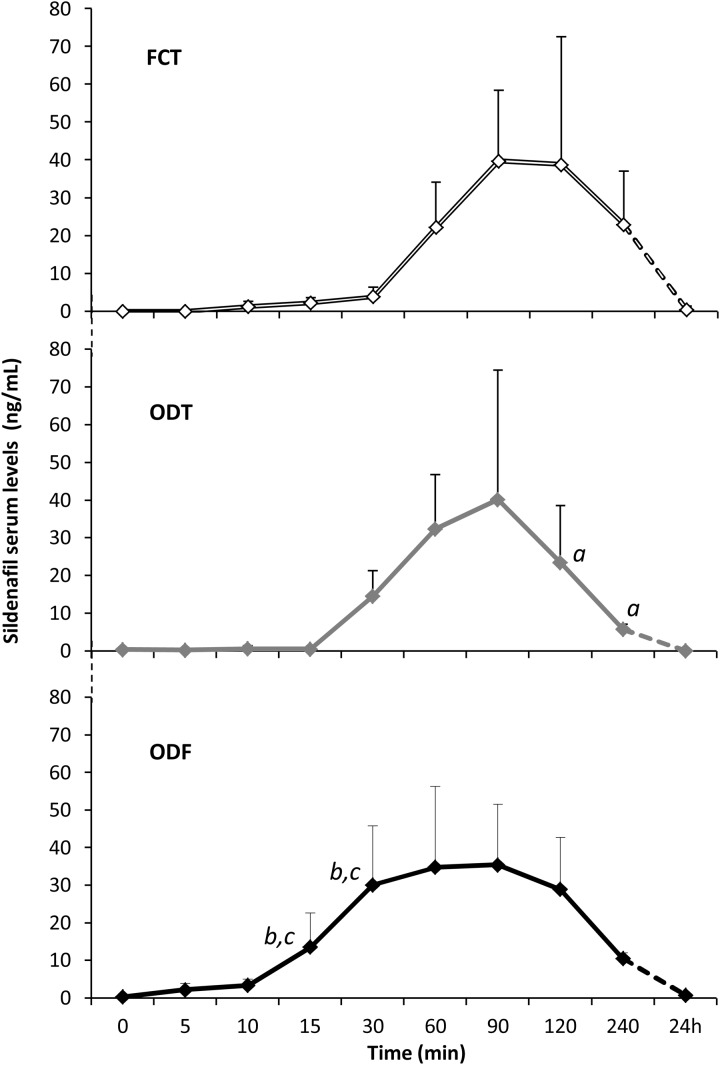
*In vivo* evaluation of serum levels of Sild, in 20 patients affected by psychogenic ED, receiving alternatively either the FCT *per os*, or the sublingual ODT, or the sublingual ODF as detailed in the section “Materials and Methods.” Data are reported as mean values ± standard deviation Significance: ^a^*P* < 0.05 vs. FCT; *^b^P* < 0.01 vs. FCT; *^c^P* < 0.05 vs. ODT.

Twenty male subjects affected by psychogenic DE accepted to receive alternatively FCT, ODT, or ODF, separated by a week of wash out. As detailed in the section “Materials and Methods,” FCT was classically swallowed to allow the classical *per os* administration while ODT and ODF were maintained under the tongue for 15 min to promote the sublingual route. Clinical characteristics of patients are reported in **Table 1**. Serum levels of Sild corresponding to the three dosing conditions are reported in **Figure 3**. A considerable variability featured the drug serum profile of the three formulations. Compared to FCT, ODT showed an apparent early increase of Sild serum levels; however, this trend was not statistically significant. On the other hand, a faster decay of Sild levels at 120 and 240 min from dosing was observed for the ODT formulation (respectively, *P* = 0.044 and *P* = 0.024 vs. FCT).

**Table 1:** Clinical characteristics of the study participants (*N* = 20).

Parameter	Mean value ±*SD*
Age (years)	31.4 ± 5.7
BMI (kg/m^2^)	26.2 ± 4.5
Waist circumference (cm)	97.4 ± 6.6
IIEF-15 (score)	19.2 ± 5.4

*Abbreviations: BMI, body mass index; IIEF-15, international index of erectile function; SD, standard deviation*.

The drug serum profile of the ODF formulation showed a faster increase of Sild levels compared to both FCT (*P* = 0.001 and *P* = 0.003) and ODT (*P* = 0.048 and *P* = 0.041), respectively, at 15 and 30 min from dosing. In agreement with its slow disaggregation time *in vitro* (**Figure 2B**), ODF mean concentration–time curve *in vivo* was smoother than those of FCT and ODT (**Figure 3**).

Pharmacokinetic parameters of the three formulations, obtained from the analysis of Sild serum levels, are summarized in **Table 2**. Despite no significant difference was observed among the three formulations in terms of *C*_max_, *t*_max_, and AUC_0-240__min_, ODF showed the lowest value of *C*_max_ (38.2 ± 23.7 ng/ml) and the shortest *t*_max_ (70.0 ± 24.5 min). In particular, ODF displayed a significant higher value of the AUC_0-60__min_ compared to both FCT and ODT (respectively, *P* = 0.005 and *P* = 0.043), resulting in an increased relative bioavailability of Sild within the first hour from the dosing of ODF through the sublingual route.

**Table 2:** Pharmacokinetic parameters of sildenafil formulations.

Formulation	*C*_max_ (ng/ml)	*t*_max_ (min)	AUC_0-240__min_ (ng/ml × min) [relative bioavailability vs. FCT (%)]	AUC_0-60__min_ (ng/ml × min) [relative bioavailability vs. FCT (%)]	AUC_0-120__min_ (ng/ml × min) [relative bioavailability vs. FCT (%)]
FCT	45.9 ± 27.3	95.0 ± 22.6	6180.2 ± 4155.4	448.1 ± 201.4	2556.0 ± 1393.8
			[100 ± 67.2]	[100.0 ± 44.9]	[100.0 ± 54.5]
ODT	48.8 ± 30.5	90.0 ± 26.8	4622.8 ± 1515.8	823.6 ± 337.8	2867.7 ± 1405.5
			[74.8 ± 24.5]	[183.8 ± 75.4]	[112.2 ± 54.9]
ODF	38.2 ± 23.7	70.0 ± 24.5	5897.7 ± 2638.6	1363.2 ± 699.0a,b	3530.8 ± 1874.2
			[95.4 ± 42.7]	[304.2 ± 156.0]	[138.1 ± 73.3]

*Significance: a = P < 0.01 vs. FCT; b = P < 0.05 vs. ODT. Repeated-measures ANOVA test was used to test the formulation × pharmacokinetic-parameter interaction with pairwise comparisons (Bonferroni–Holm adjusted). Abbreviations: FCT, film-coated tablets; ODT, oro-dispersible tablets; ODF, oro-dispersible film, AUC, area under the curve*.

### Adverse Drug Reactions

Results obtained from the self-administered questionnaire on the type and features of ADR experienced by the study participants are summarized in **Table 3**. Among the known ADR reported for Sild (Taylor et al., 2009), the most frequently recorded were headache, flushing, and nasal congestion. One patient reported low grade muscle pain (grade 1 at the subjective assessment scale) after the dosing of FCT. None of the patients reported altered vision, tachycardia, weakness, or altered hearing.

**Table 3:** Adverse drug reactions recorded by the study participants (*N* = 20).

	FCT			ODT			ODF		
	Cases (%)	Duration (min)	Intensity (score)	Cases (%)	Duration (min)	Intensity (score)	Cases (%)	Duration (min)	Intensity (score)
Headache	7 (35)	66.4 ± 31.2	2.9 ± 2.4	6 (30)	41.6 ± 12.7	1.8 ± 1.4	1 (5)a	30	1
Altered vision	0	//	//	0	//	//	0	//	//
Tachycardia	0	//	//	0	//	//	0	//	//
Weakness	0	//	//	0	//	//	0	//	//
Altered hearing	0	//	//	0	//	//	0	//	//
Flushing	10 (50)	75.9 ± 42.3	3.6 ± 2.1	4 (20)	57.1 ± 13.4	2.8 ± 1.1a	4 (20)	25.0 ± 9.2a,b	1.3 ± 0.7a,b
Muscle pain	1 (5)	120	1	0	//	//	0	//	//
Nasal congestion	8 (40)	51.3 ± 27.1	3.3 ± 1.8	5 (25)	47.4 ± 11.3	2.9 ± 1.6	6 (30)	28.2 ± 10.4a,b	1.4 ± 0.9a,b

*Significance: a = P < 0.05 vs. FTC; b = P < 0.05 vs. ODT. The proportion of ADR was compared with χ^*2*^ exact test. ANOVA was used to test the duration and intensity of ADR by pairwise comparisons (Bonferroni–Holm adjusted). Abbreviations: FCT, film-coated tablets; ODT, oro-dispersible tablets; ODF, oro-dispersible film; min, minutes*.

Compared to FCT as the reference formulation, ODT showed unvaried prevalence of ADR. However, the personal perception of flushing intensity was significantly lower (*P* = 0.031). On the other hand, ODF showed a reduced prevalence of headache compared to FCT (*P* = 0.043). Furthermore, the duration and intensity of flushing and nasal congestion were perceived at lower levels compared to both FCT (respectively, *P* = 0.011 and *P* = 0.015) and ODT (respectively, *P* = 0.026 and *P* = 0.037).

## Discussion

In this study, we provide evidence that the sublingual route of Sild administration associates with an increased early drug bioavailability and improved tolerability profile. This evidence is supported by both *in vitro* investigations, showing that the Sild formulation is featured by a longer time to disaggregation but significantly favored trans-mucosal absorption, and *in vivo* evidences.

Phosphodiesterase inhibitors are the first choice therapeutic option for the treatment of ED; however, side effects experienced by patients are acknowledged as the most prevalent reason for the discontinuation of the therapy (Corona et al., 2016). Modification of pharmacokinetics through the design of novel drug formulation may represent an attractive strategy to improve the safety/efficacy profile of the drug itself (Mehrotra et al., 2007). To this regard, the 2013 expiry of the patent on Sild citrate in several European countries allowed the opportunity to release of a number of new formulations of Sild. It should be noted that most of the Sild formulations available on the market today are actually approved as bioequivalent forms of the original FCT (Leoni et al., 2013). Interestingly, orally disintegrating formulations, featured by rapid disaggregation in the patient’s mouth without the need for swallowing with water, have been developed (Goel et al., 2008). These novel formulations find improved compliance in those populations of patients with difficulty in swallowing conventional solid dosages, such as children, geriatric patients, and dysphagic patients. In addition, by providing higher oral drug availability, orally disintegrating formulations may represent valued systems to favor trans-mucosal, and in particular sub-lingual, absorption (Kathpalia and Gupte, 2013). Particularly in Italy, two orally disintegrating formulations of Sild are officially available on pharmacy facilities: the ODTs and the ODFs.

In this study, we aimed to evaluate the possible pharmacokinetic variations, and the corresponding pattern of ADR, deriving from favoring the trans-mucosal absorption of Sild through the sub-lingual administration of the two available orally disintegrating formulations of the drug. Results were compared with the original formulation of Sild, namely the FCTs. Interestingly, we found a strict correlation, both *in vitro* and *in vitro*, between the bio-technological properties and the PKs of the different formulations. In particular, in spite of an equal drug dosage (50 mg) and slightly reduced rate of drug release in the dissolution test, ODF was characterized by a greater time to disaggregation and higher rate of permeation in the trans-mucosal model on the one hand, and by an increased drug bio-availability within the first hour from dosing, on the other hand. We might then hypothesize that sub-lingual Sild absorption is favored by a prolonged drug-mucosa contact. Indeed, this phenomenon is generally pursued for oral trans-mucosal drugs, where the use of mucoadhesive polymers is recommended to assure prolonged period of contact between the formulation and the oral mucosa (Lam et al., 2014), in particular from those drugs, like Sild, affected by low water solubility (Zayed et al., 2012; Sun et al., 2014). These preliminary results are also suggestive of a possible improvement of the clinical efficacy for this class of drugs compared to original formulations. Further studies are warranted to clarify these aspects.

The most striking evidence of the present study is a significant reduction in the ADRs of sub-lingual administration of ODF, compared to ODT and FCT administered at the same dosage, which in turn may result in increased patients’ compliance. This evidence can be interpreted in the light of the principle that the incidence of a drug side effect increases by increasing serum levels and the exposure time to the drug itself (Gupta et al., 2005; Taylor et al., 2009). In spite of an unvaried global bio-availability compared to the other formulations analyzed, the sub-lingual administration of ODF showed a lower value of *C*_max_ and a shorter *t*_max_, resulting in a significantly higher proportion of Sild bio-available within the first hour from dosing. Thus, we might speculate that the sub-lingual administration of ODF combines an efficient absorption of the drug with the achievement of average lower serum levels of Sild and a consequent lower risk of ADRs. However, we acknowledge the low sample size as the main drawback of the study. Further investigation on larger cohorts and with different dosages are required to confirm this issue.

In conclusion, in this study we report that sublingual administration of Sild ODF improves the drug tolerability profile through the likely involvement of a modified pharmacokinetic compared to generator FCTs. Although ODF was not originally conceived for this purposes, this represent an attractive strategy to ameliorate the patient’s compliance to therapy. We are developing further comparative studies to assess the clinical efficacy in psychogenic and organic ED patients.

## Author Contributions

LDT and CF coordinated the study and drafted the manuscript. MDRP evaluated and enrolled the patients. EF performed *in vitro* analysis. SDA performed the serum analysis. RP performed the pharmacokinetics comparison. RP, NR, and AG critically revised and finalized the manuscript.

## Conflict of Interest Statement

The authors declare that the research was conducted in the absence of any commercial or financial relationships that could be construed as a potential conflict of interest.

## Footnotes

1 http://www.aifa.gov.it/en/content/reporting-adverse-reaction

## Supplementary Material

FIGURE S1 Representative images of the Tablet Disintegration Tester **(A)** and the Dissolution Apparatus **(B)** used for disaggregation test and dissolution test, respectively.

FIGURE S2 Details of the mobile phase gradient used for the high performance liquid chromatography-tandem mass spectrometry (HPLC-MS/MS) analysis of serum levels of sildenafil (Sild). Representative chromatograms and mass spectra of both Sild at the internal standard benzanilide in real samples are reported **(B)**. **(C)** Representative image of residual oro-dispersible film (highlighted by a discontinuous line), persisting after the permeation test in the device used for the *in vitro* estimate of trans-mucosal absorption through a cellulose acetate membrane.

DATA SHEET S1 Adverse drug reaction questionnaire.
