# Supplementary figures and images for: Sublingual Administration of Sildenafil Oro-dispersible Film: New Profiles of Drug Tolerability and Pharmacokinetics for PDE5 Inhibitors
Source: Front Pharmacol. 2018 Feb 6;9:59. doi: 10.3389/fphar.2018.00059 (PMC5808105; doi:10.3389/fphar.2018.00059)

A

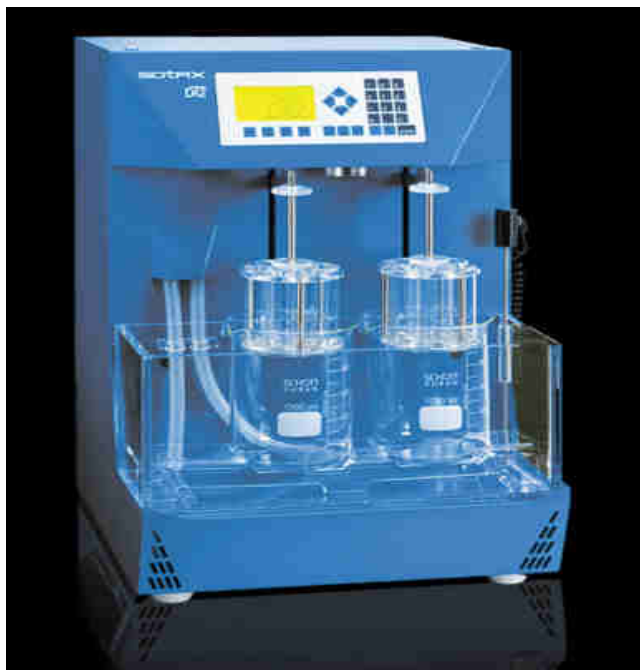

B

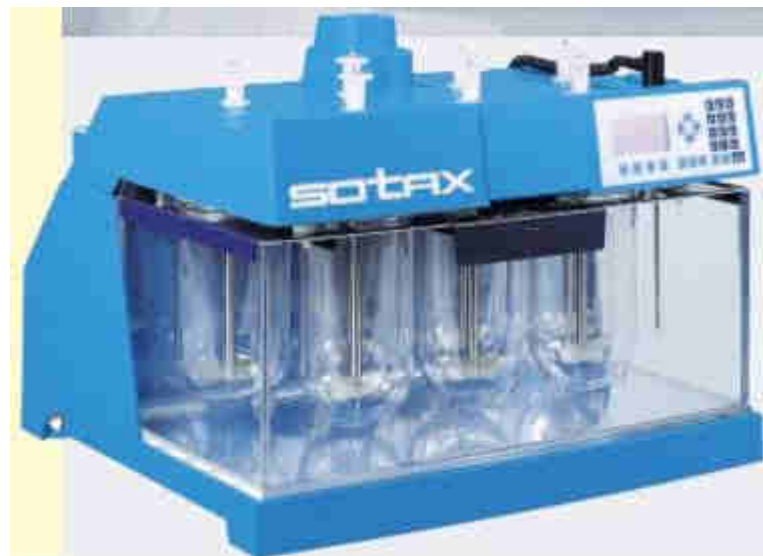

Supplemental Figure S1

Supplement: FIGURE S1 — Representative images of the Tablet Disintegration Tester (A) and the Dissolution Apparatus (B) used for disaggregation test and dissolution test, respectively. [file Image_1.PDF]
