# Supplementary material for: Sublingual Administration of Sildenafil Oro-dispersible Film: New Profiles of Drug Tolerability and Pharmacokinetics for PDE5 Inhibitors
Source: Front Pharmacol. 2018 Feb 6;9:59. doi: 10.3389/fphar.2018.00059 (PMC5808105; doi:10.3389/fphar.2018.00059)
Supplement: FIGURE S2 — Details of the mobile phase gradient used for the high performance liquid chromatography-tandem mass spectrometry (HPLC-MS/MS) analysis of serum levels of sildenafil (Sild). Representative chromatograms and mass spectra of both Sild at the internal standard benzanilide in real samples are reported (B). (C) Representative image of residual oro-dispersible film (highlighted by a discontinuous line), persisting after the permeation test in the device used for the in vitro estimate of trans-mucosal absorption through a cellulose acetate membrane. [file Image_2.PDF]

A

Time (min)	% Acetonitrile	% Water + 0,1% Formic acid	Flow (µl/min)
0	45	55	300
5	100	0	300
6,5	100	0	300
7,0	45	55	300
9,5	45	55	300

B

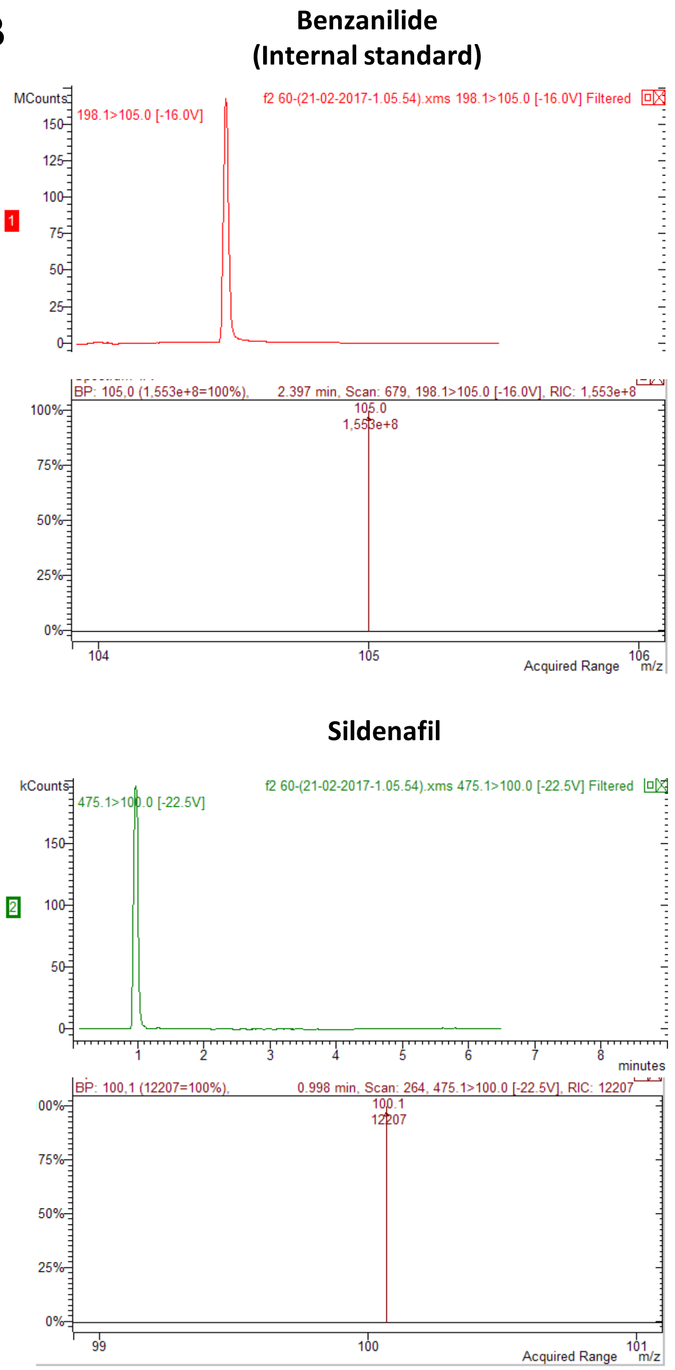

C

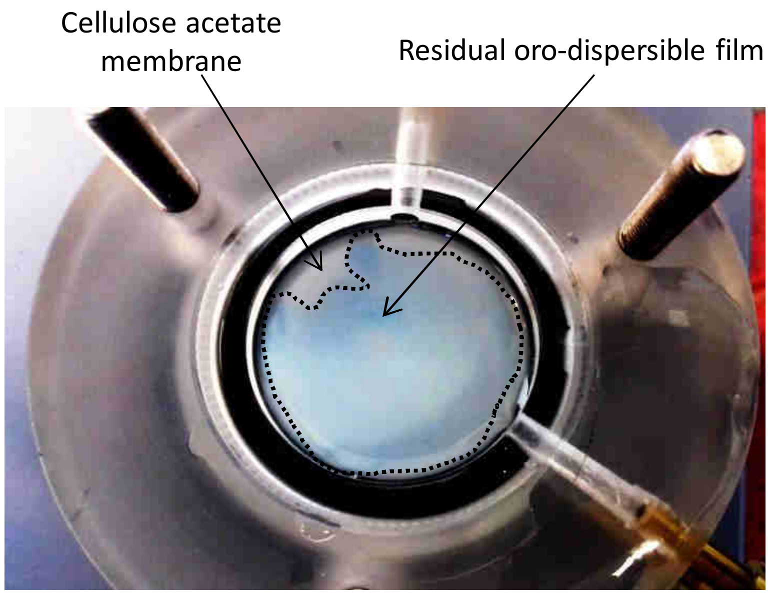

Supplemental Figure S2
