# Supplementary material for: Sublingual Administration of Sildenafil Oro-dispersible Film: New Profiles of Drug Tolerability and Pharmacokinetics for PDE5 Inhibitors
Source: Front Pharmacol. 2018 Feb 6;9:59. doi: 10.3389/fphar.2018.00059 (PMC5808105; doi:10.3389/fphar.2018.00059)
Supplement: DATA SHEET S1 — Adverse drug reaction questionnaire. [file Data_Sheet_1.doc]

**ADVERSE DRUG REACTION QUESTIONNAIRE**

PATIENT’S IDENTIFICATION NUMBER:……………………

1

- Date of administration l__l__l/l__l__l/l__l__l__l__l
- Time of administration l__l__l:l__l__l
- Drug administered l_______________l Dosage l____________l
- The drug was assumed in fasting state? ○ YES ○ NO
- The drug was assumed with alcohol ○ YES ○ NO

• Did you experience an adverse reaction after assuming the drug?

○ YES ○ NO

- In case you answered YES in the previous item, how would you classify the adverse reaction/s? (more than one is accepted)

○Headache ○Weakness ○Nasal congestion

○Altered vision ○Altered hearing ○Muscle pain

○ Tachycardia ○Flushing ○Other:…………………

- How would score the overall intensity of the adverse reaction/s from 1 (weak) to 5 (very intense),

1○ 2○ 3○ 4○ 5○

- • How long was the overall duration of adverse reaction/s?

l______l (minutes, approximately)

- In general, did the adverse reaction discourage the further use of the drug?

○ YES ○ NO
